# Application of Electromigration Techniques in Clinical Bioanalysis of Heteroatom-Containing Agents

**DOI:** 10.3390/ijms262211019

**Published:** 2025-11-14

**Authors:** Marián Masár, Peter Troška, Josef Jampilek, Massoud Kaykhaii

**Affiliations:** Department of Analytical Chemistry, Faculty of Natural Sciences, Comenius University Bratislava, Ilkovicova 6, SK-842 15 Bratislava, Slovakia; marian.masar@uniba.sk (M.M.); peter.troska@uniba.sk (P.T.); massoud.kaykhaii@uniba.sk (M.K.)

**Keywords:** capillary electrophoresis, bioanalysis, body fluids, biomarkers, electromigration techniques, clinical diagnostics

## Abstract

The bioanalysis of body fluids plays a crucial role in clinical diagnostics, pharmaceutical research, forensic science, and biomarker discovery. Traditional chromatographic techniques are widely used in clinical laboratories but are often costly and time-consuming. Capillary electrophoresis (CE) and its modifications, such as capillary zone electrophoresis, isotachophoresis, and micellar electrokinetic chromatography, have emerged as efficient, cost-effective, and miniaturized alternatives for analyzing small organic and inorganic molecules in biological fluids. This paper deals with the applications of CE-based electromigration techniques in the determination of various analytes in urine, blood, saliva, and cerebrospinal fluid. The study further discusses the advantages and limitations of different detection methods, including ultraviolet-visible spectroscopy, laser-induced fluorescence, mass spectrometry, conductivity, and amperometric detection. A focus is given to the identification and quantification of amino acids and their metabolites as potential biomarkers for metabolic and degenerative disorders. The work highlights recent advancements in CE methodologies and their potential to enhance sensitivity and selectivity in bioanalytical applications.

## 1. Introduction

Biological samples mostly consist of body fluids. Among them, the most frequently analyzed are urine, blood, saliva, and cerebrospinal fluid (CSF). Apart from water, these samples contain many inorganic and organic compounds. It is necessary to determine the extent to which changes in the concentrations of these compounds can predict various types of diseases, both inborn and those that gradually manifest over time. Inorganic and organic ions present in every human’s fluid constitute a large group of such substances. The organic substances that are integrally significant in body fluid analysis are amino acids and peptides, which can indicate various developmental and metabolic disorders or diseases of the tested individual.

Urine and saliva sampling are non-invasive. They can be performed at home in the absence of a healthcare specialist. Different small organic and inorganic ions [1], as well as small biological molecules, are detected in such samples. Analysis of blood samples is considered a more invasive sampling method, which requires the presence of a healthcare worker to perform the sampling. The most invasive sampling method is CSF sampling, which requires the patient to be under general anesthesia.

Chromatographic techniques combined with different detectors are most often implemented in clinical laboratories. These techniques are cumbersome and expensive. Therefore, researchers are striving to develop new, cheaper separation techniques. A suitable alternative to chromatographic separation techniques in terms of cost-effectiveness is capillary electrophoresis (CE) and its various alternatives, such as capillary zone electrophoresis (CZE), isotachophoresis (ITP), micellar electrokinetic chromatography (MEKC), and their miniaturized versions, known as microchip electrophoresis (MCE), performed on a glass or polymer microchip [2]. Since electromigration methods are based on the separation of substances in a uniform electric field, these techniques are primarily suitable for the determination of ionizable substances in the aforementioned body fluids.

At this point, it should be noted that CE in this work was used as a general (umbrella) term for analytical techniques for ion separation in narrow capillaries using an electric field. In this context, CE encompasses a range of electroseparation techniques, each of which exhibits considerable potential for enhancing analytical sensitivity and/or overall separation selectivity in clinical bioanalysis, but differs primarily in the number and composition of the electrolytes used. These techniques employ narrow-bore capillaries to achieve high-efficiency separations and include, but are not limited to, the following:

- CZE, which involves the introduction of the sample plug into a single, uniform background electrolyte (BGE);
- ITP in which the sample is introduced between two distinct electrolyte systems, leading electrolyte (LE) and terminating electrolyte (TE), facilitating sample stacking and separation based on differential ionic mobilities;
- isoelectric focusing (IEF), which utilizes a combination of three distinct electrolytes to establish a pH gradient, whereupon analytes migrate and concentrate based on their respective isoelectric points (pI), etc.

Consistent with this classification framework, i.e., the criterion of the number of electrolytes used for the separation, MEKC is classified as a variant of CZE, wherein separation is achieved in the BGE under micellar pseudostationary phase conditions.

The most common way to analyze biological samples using electromigration methods is spectrophotometric determination, such as LIF or UV-visible detection. However, not all analyzed substances absorb electromagnetic radiation at a certain wavelength. Therefore, in this case, complex sample preparation is required, where a derivatization reagent is added to the sample. The reagent binds with the analytes and thus changes their physical and chemical properties. Higher sensitivity and lower detection limits (trace and ultra-trace amounts) of an analytical method can be achieved by this means. Universal conductivity detection, which does not require any modification of the sample or analytes, has been applied as well. However, the values obtained between detections may not be as satisfactory as in the case of selective detection methods.

As will be discussed in this paper, electromigration methods, particularly CZE, MEKC and ITP, have been extensively applied to analyze various substances in body fluids. These methods are valued for their ability to handle complex biological matrices and provide high-resolution separations.

The latest work closely related to this subject was published in 2005 [3]. A chapter published in 2021 focused on the main modes of separation in CE, primarily discussing the instrumental aspects while also addressing some typical bioanalytical applications [4]. Therefore, the goal of this paper is to summarize scientific publications dedicated to the analysis of body fluids for the determination of selected groups of substances by electromigration methods using different detection techniques.

## 2. Bioanalysis

**Figure 1:**
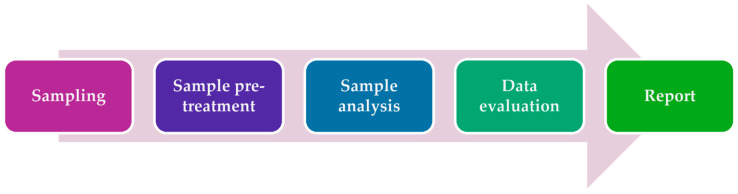
Overview of the bioanalytical workflow: key steps from sample collection to data interpretation.

The term “bioanalysis” is closely related to the analysis of body fluids. The term has been used since the 1930s, predominantly in connection with the analysis of pharmaceuticals in body fluids to define the pharmacokinetics of a medicine [5,6,7]. Bioanalytical methods are not restricted to the analysis of small inorganic and organic molecules, but also include the analysis of biopolymers, such as proteins and peptides [8]. Since bioanalysis is an important scientific discipline utilized for the development of pharmaceuticals, forensic analysis, doping control, and the identification of biomarkers for various medical conditions, it is widely implemented in pre-clinical, clinical, toxicological, and forensic laboratories [9,10]. The validation of bioanalytical methods is a critical step in bioanalysis. Through validation, it is possible to quantify different types of analytes present in body fluids. Generally, the bioanalytical approach includes several steps, from sampling to reporting the obtained data (Figure 1).

### Body Fluids

A golden standard in clinical, forensic, and toxicological science is the analysis of urine and blood serum (plasma). However, in recent years, other types of body fluids have been in the foreground, such as CSF, saliva, sweat, and breast milk [11].

From the biomedical point of view, urine is a secondary liquid product of a living organism’s metabolism. Approximately 95% of urine is water, and 5% is urea, chlorides, sulfates, potassium, sodium, calcium, and other organic and inorganic components. It is one of the most frequently analyzed body fluids because the sampling and collection of urine is easy and non-invasive, and can be performed in the absence of a healthcare specialist. It is important to choose the correct time for urine sampling because the nature and amount of separate compounds in a urine sample vary throughout the day. From the medical point of view, there are three types of urine samples: first morning samples, overnight samples, and 24 h samples [12,13].

Saliva is an aqueous secretion of the salivary glands, comprising a complex mixture of organic and inorganic substances. The principal constituents of saliva are enzymes that catalyze the cleavage of complex matrices in the consumed food [14]. The analysis of saliva has become a useful tool for diagnosing diseases based on its origin, composition, functions, and interactions with other organ systems. Moreover, saliva collection is easy and non-invasive, saliva samples are easily storable, and saliva collection is cheaper than blood sampling [14].

The second most frequently used liquid for analysis is blood, namely, blood serum and blood plasma. The difference between serum and plasma is the presence or absence of coagulants. Blood serum analysis has been applied for the identification of various endocrinological, cardiovascular, and inflammatory diseases [15,16,17].

CSF is the solution filling both the spinal cord and the cranial cavity. It plays a significant role in the regulation of liquids and nutrient transport in the central nervous system. Since the composition of CSF directly depends on the speed of metabolome production in the brain, it is possible to obtain information on diseases of the central nervous system, such as brain injury, Parkinson’s and Alzheimer’s diseases, and others, by analyzing the fluid. Collection of CSF is the most complicated; hence, it is usually performed by a qualified medical worker using lumbar puncture [18].

## 3. Capillary and Microchip Analysis of Biosamples

**Figure 2:**
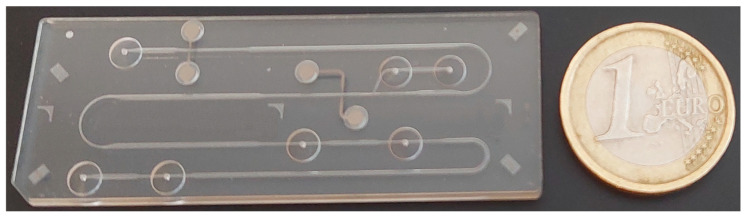
Photograph of a polymethyl methacrylate microchip with coupled channels and integrated conductivity sensors used for electrophoretic analysis.

**Figure 3:**
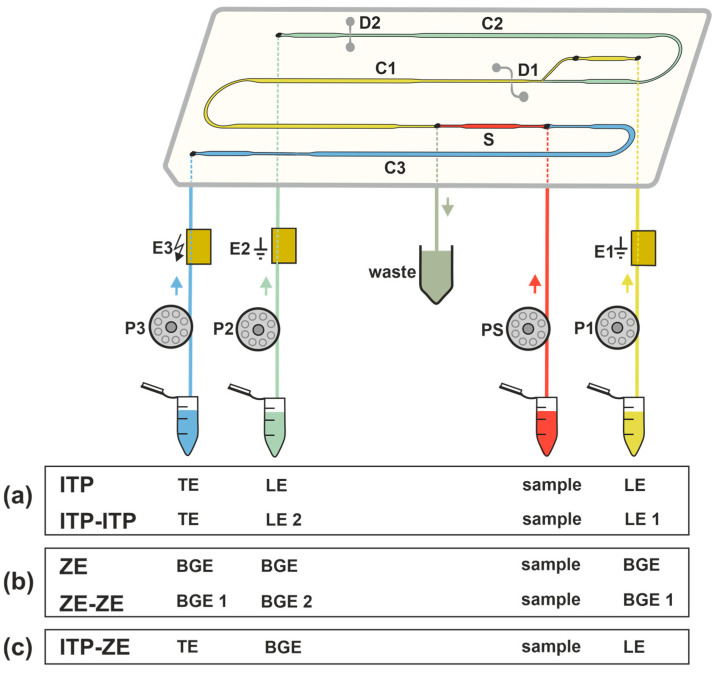
Diagram of MCE instrumentation: electrolyte configurations in separation channels for (**a**) ITP and tandem ITP-ITP; (**b**) ZE and tandem ZE-ZE and (**c**) combined ITP-ZE modes. C1, C2—first and second separation channels; C3—auxiliary channel; S—sample channel; D1, D2—conductivity sensors; E1, E2—driving electrodes for the C1 and C2, respectively; E3—driving electrode permanently connected to high-voltage power supply; P1, P2, P3, PS—peristaltic micropumps for filling microchip channels with electrolytes and sample; LE—leading electrolyte; TE—terminating electrolyte; BGE—background electrolyte.

CE and MCE are electromigration separation analytical methods. Their principle is the movement of charged particles under the influence of a high-intensity direct electric field. The separation of charged substances contained in the sample occurs due to different electrophoretic mobilities in a given electrolytic system. The basic electrophoretic techniques, based on which it is possible to perform separations of charged substances in CE and MCE, are zone electrophoresis (ZE), ITP, isoelectric focusing, and moving boundary electrophoresis. The advantages of CE and MCE are simple instrumentation, high separation efficiency, short analysis time, and minimal sample and reagent volumes [19,20]. These properties are particularly interesting from the point of view of biomedical applications, which require a fast analytical response and are often limited by the amount of collected sample. In CE, the separation space is a capillary, which is most often made of fused silica with an internal diameter of 25–100 µm and a length of 50–100 cm. Microchips for MCE were initially made of etched glass and usually had only two channels. The sample was diluted into one, and the separation was performed in the other. Over time, new types of microchips were developed that were able to improve the separation efficiency. In addition, new technological processes began to be used for the production of microchips. In terms of price requirements, microchips began to be produced from polymeric materials such as polydimethylsiloxane (PDMS), polymethyl methacrylate (PMMA), polyvinyl chloride (PVC), and others. More complex microchips have coupled separation channels (see Figure 2) that use a column switching system, which allows achieving higher separation efficiency [21]. It is also possible to perform various electrophoretic techniques by simply changing the electrolyte solutions in the separation channels (Figure 3). An example is the MCE enantioseparations of Trp by ITP and ITP-ITP [22] and MCE bioanalyses of CSF, which have been performed using ZE [23,24], ZE-ZE [23,24] and ITP-ZE [25].

### 3.1. Amino Acids

Amino acids are low-molecular-mass chemical substances readily soluble in water. From a chemical point of view, they are composed of at least one amino group and one carboxylic group. Both groups may easily dissociate, and their charge depends only on the pH of the buffer solution used. It is important to determine amino acids in different types of body fluids since they can serve as biomarkers for various degenerative diseases, inborn disorders, or dysfunctions of some organs. Amino acid determination is possible by means of both CZE and MEKC. Amino acids are most often determined by coupling MEKC with LIF, and the analysis itself requires sample pre-treatment by derivatization [26]. The combination of CZE and electrospray ionization-tandem mass spectrometry (ESI-MS^2^) allows for detailed profiling of all 20 essential amino acids, as a 220 nM LOD for arginine can be obtained [27]. Furthermore, capacitively coupled contactless conductivity detection (C^4^D) is also applicable for the simultaneous determination of 20 essential amino acids [28].

A beneficial method of determination of amino acids in CSF is the combination of CZE coupled with MS and CZE with C^4^D, albeit LOD values in the latter case are somewhat higher [29,30]. Nevertheless, micro electroextraction has been proven to greatly decrease the LODs in CZE-MS measurements [31]. Urine, due to its easy non-invasive collection and typically very simple pre-treatment (limited to dilution with water), is a popular body fluid to determine various analytes and CE can be applied to determine all proteinogenic amino acids in urine [30,32]. Conversely, determination of amino acids in whole blood, blood plasma or tears may require more complex sample preparation, such as dialysis using selective membranes, as proposed by Tůma [28]. LODs can be decreased greatly by means of extraction techniques as a pre-treatment method. In these terms, one of the most effective extraction techniques for the determination of amino acids in urine is two-phase micro electroextraction, as demonstrated by Oedit et al. who managed to reach LODs for eight proteinogenic amino acids in the nanomole range [31]. Reduced levels of branched chain amino acids (valine, leucine, isoleucine) in blood may be a sign of pancreatic cancer, which is usually hard to detect at early stages due to lack or slightness of symptoms. Therefore, controlling concentrations of those amino acids in patients with cancer cachexia (a progressive syndrome characterized by muscle deterioration and chronic fatigue) can be helpful for diagnosing pancreatic cancer, as demonstrated by Tůma et al., who reached LODs lower than 1 μM for Val, Leu, and Ile [33].

A new type of covalently coated capillary based on polyacrylamide and (3-acrylamidopropyl)trimethylammonium chloride was used to determine plasma levels of valine, isoleucine, leucine, alanine and glutamine by CZE-C^4^D [34]. For physiological studies, rapid and sensitive determination of the branched-chain amino acids valine, isoleucine and leucine in human plasma was also performed by CZE-C^4^D [35]. Zhu et al. developed a new alternative injection strategy for flow-gate CZE to enable its quantitative capability. Nineteen amino acids were determined in urine using MEKC with LIF detection and the LOD for these amino acids was estimated to be below 5.0 nM [36]. Urine samples were also analyzed by CZE-MS. The LOD for 27 amino acids ranged from 0.63 to 29.0 µM [37]. The neurotransmitters γ-aminobutyric acid (GABA), glycine (Gly) and glutamate (Glu) were determined in periaqueductal gray matter microdialysates using a novel variant of large-volume sample injection in CZE-C^4^D. This approach allowed LOD levels in the range of 9–15 nM to be achieved [38].

Sarcosine (Sar) metal-coded hydrogel magnetic molecularly imprinted polymer (Hydro-MeC-MMIP) was synthesized and applied to on-column derivatization CE for the extraction, preconcentration, and quantification of urinary Sar, a potential biomarker of prostate cancer [39]. The determination of the amino acids homocysteine (Hcy) and cysteine (Cys) is important for biological, clinical, and pharmaceutical research. These compounds were quantified in human blood plasma and urine using CZE coupled with liquid–liquid extraction (LLE) and UV detection. After derivatization of the analytes with 1,1′-thiocarbonyldiimidazole, the samples were purified by chloroform and acetonitrile (ACN) extraction [40].

**Figure 4:**
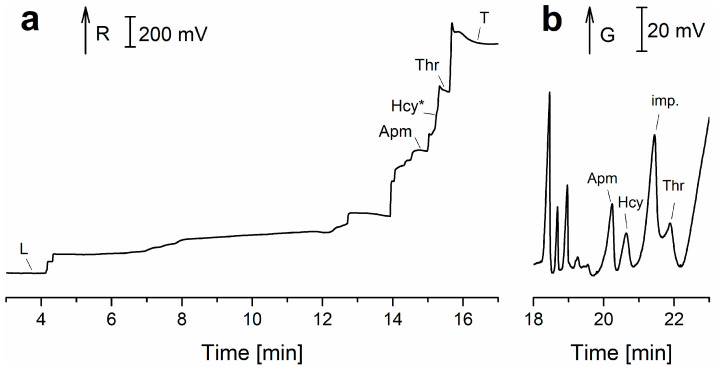
Microchip ITP-ZE bioanalysis of urine after SPME pre-treatment obtained from contact conductivity detector placed in (**a**) 1st and (**b**) 2nd channel. ITP-ZE separations were performed in the electrolyte system consisting of LE: 10 mM hydrochloric acid, 20 mM *bis*-*tris* propane, 0.1% *w*/*v* MHEC (pH 9.1); TE: 10 mM glycine, 10 mM *bis*-*tris* propane, 0.1% *w*/*v* MHEC (pH 9.4); and BGE: 20 mM β-alanine, 25 mM *bis*-*tris* propane, 0.1% *w*/*v* MHEC (pH 9.8). MHEC—methylhydroxyethylcellulose; L—leading ion; T—terminating ion; Apm—aminopimelate; Hcy—homocysteine; Hcy*—migration position of Hcy in ITP; Thr—threonine; imp.—impurity; R—resistance; G—conductivity. Modified from [47], University of Pannonia/De Gruyter Open, 2018.

**Figure 5:**
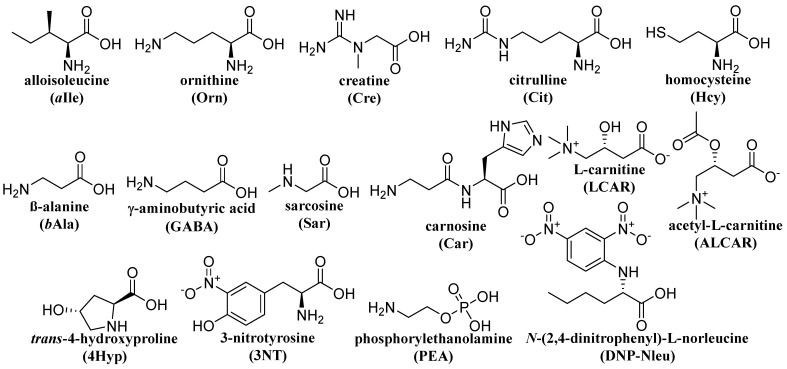
Structures of uncommon amino acids and related compounds.

The advantages of CZE with online ITP sample pretreatment in a column-coupled (CC) CE device for the separation and determination of enantiomers present in multicomponent ionic matrices were demonstrated for d-, l-tryptophan (Trp) [41]. A similar ITP-CZE combination approach has been used for the determination of 2,4-dinitrophenyl-labeled amino acids in urine [42]. In general, the importance of ITP pretreatment focuses on (i) concentration of analytes; (ii) post-column sample clean-up; and (iii) injection of analytes, concentrated in a narrow band, onto the CZE column for their separation in the CZE phase [43]. Trp enantiomers as model analytes were separated by ITP on a PMMA microchip with coupled separation channels and contact conductivity detection (CD) (Figure 3a). Single-column ITP, ITP in the tandem-coupled column, and concentration-cascade ITP in the tandem-coupled columns were used in this study [22]. The same microchip with coupled channels and CD was used for the ZE enantioseparation (Figure 3b) of non-derivatized aspartic acid (Asp) and glutamic acid (Glu). Vancomycin added to BGE served as a chiral selector. For a comparative study, CZE with indirect UV detection and identical BGE composition was used [44]. MCE of l-carnitine (LCAR) and acetyl-l-carnitine (ALCAR) was performed in milk samples such as breast milk. LOD values of 0.2 μM and 0.6 μM were obtained for LCAR and ALCAR using CD [45]. For the detection of 3-nitrotyrosine after ZE separation in urine, a CD was used in the first separation channel of the CC microchip and a visible photometric detector (Vis) in the second separation channel [46]. A bioanalysis using the ITP-ZE combination on a microchip (Figure 3c) was used for the sensitive determination of Hcy in urine and saliva. Chlorides and sulfates naturally present in the analyzed body fluids were removed by solid-phase microextraction (SPME) pretreatment. The ITP performed in the first separation channel on the microchip enabled preconcentration of the analyte and other sample constituents before the ZE separation step. However, the close migration configuration of constituents during the ITP stage can limit the resolution between the analyte and matrix constituents, especially on microchips with short separation channels. Therefore, discrete spacers aminopimelate and threonine were used to define the fraction of the sample transferred to the second ZE channel (Figure 4). The LOD for Hcy was 1.4 μM [47] (see Table 1, summarizing the above-mentioned methods). The formulas of selected, less common amino acids mentioned in Table 1 are shown in Figure 5.

### 3.2. Peptides

Peptides are macromolecules comprising 2–50 amino acids connected by peptide bonds. The presence of certain peptides in body fluids can serve as a biomarker of diseases, e.g., malignant and benign tumors. They are the most widely represented and most important biomolecules. Peptides can act as neurotransmitter hormones, immunomodulators, antibiotics, catalysts and inhibitors of biochemical reactions, and toxins. Thus, they play an important role in many natural processes in living organisms [48].

A wide range of analytical methods is used for the determination of peptides in body fluids. However, analysis of peptides in biological samples is usually complicated because of the complexity of the samples. The presence of peptides in body fluids can serve as an indicator of certain diseases. For example, a hydroxyproline-containing peptide was proposed as a biomarker of bone resorption [49]. Various electrophoretic techniques have been applied to determine peptides associated with atherosclerosis, rheumatoid arthritis, adult respiratory distress syndrome (glutathione) [50,51], chronic pain (opioid peptides) [52], chronic kidney disease [53], Alzheimer’s disease [52,54], osteoporosis [55].

Glutathione is an important low-molecular-weight thiol naturally present in human fluids, and its accurate determination is essential for biochemical research. For its enrichment and detection, hollow-fiber liquid-phase microextraction combined with CZE and amperometric detection (AD) achieved up to 471-fold enrichment from saliva without derivatization [50], while a complementary approach using gold nanoparticles capped with Tween 20 enabled rapid extraction of multiple thiols, including glutathione, with enrichment factors up to 2067-fold [51]. Both techniques provided high sensitivity, good recoveries, and simple sample preparation, offering effective tools for the analysis of biological fluids. The simultaneous determination of glutathione and glutathione disulfide concentrations in bovine blood samples was performed in a CC instrument with CD using a capillary ITP. The concentration of the analytes was realized by a concentration cascade, when different concentrations of a leading ion were used in the coupled columns [56].

The nanoliter valve SPE CZE-MS system was introduced to improve sample handling and preconcentration compared to conventional unidirectional designs. Demonstrated for opioid biomarkers in plasma, it achieved up to 200-fold lower LODs, better peak efficiencies, and greater tolerance to complex matrices, making it a versatile tool for bioanalysis [52]. Another study reported an ITP-ESI-MS method that enabled direct detection and quantification of several amyloid-β peptides in cerebrospinal fluid without immunocapture pre-treatment, achieving sensitivity down to 50 pM. By coupling ITP preconcentration with MS, the approach provided reliable measurements and accurate determination of peptide ratios in patient and control samples [54].

Zhang et al. developed to accurately and selectively determine free prolyl compounds in unhydrolyzed urine by blocking primary amines with *o*-phthalaldehyde and selectively derivatizing secondary amines with 4-fluoro-7-nitro-2,1,3-benzoxadiazole, followed by direct analysis on a flow-gated CE system. Six prolyl compounds (Pro-Hyp, Pro-Pro, Pro-Gly, Pro-Leu, Hyp, and Pro) were separated within 30 s (over 60-fold faster than HPLC) with LODs down to 20–60 nM, offering a simplified and efficient protocol for analyzing prolyl dipeptides and Hyp in biological fluids [55].

**Figure 6:**
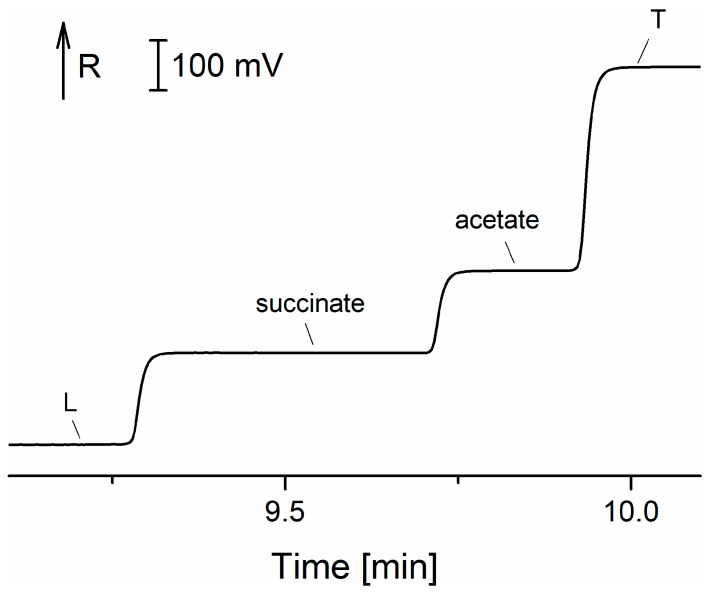
Microchip ITP bioanalysis of buserelin acetate pharmaceutical preparation. ITP separations were performed in the electrolyte system consisting of LE: 10 mM hydrochloric acid, 20 mM histidine, 0.1% *w*/*v* MHEC (pH 6.1); and TE: 15 mM caproic acid, 25 mM histidine, 0.1% *w*/*v* MHEC (pH 6.0). MHEC—methylhydroxyethylcellulose; L—leading ion; T—terminating ion; R—resistance. Modified with permission from [61], Springer Nature, 2016.

**Figure 7:**
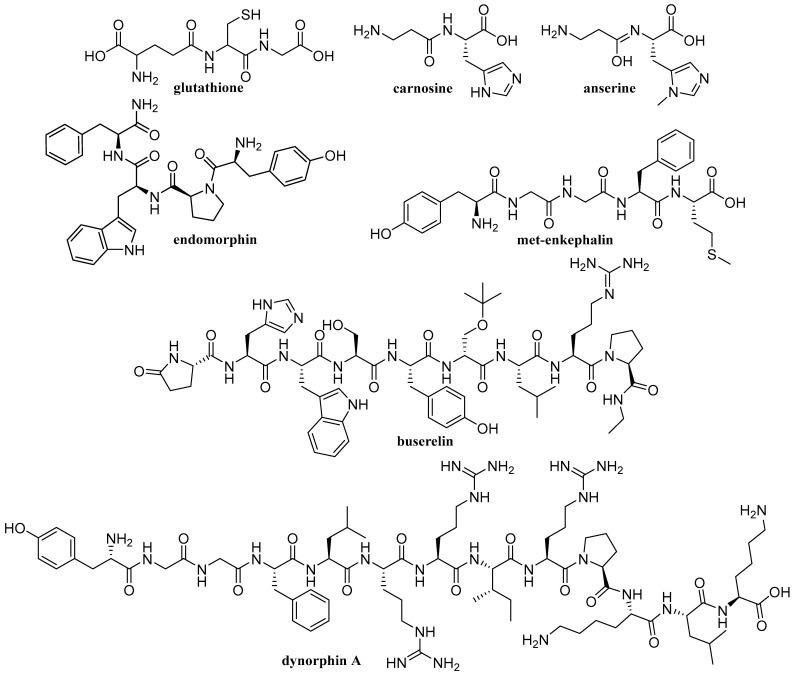
Structures of selected peptides.

Staňová et al. were focused on the bioanalysis of the nanopeptide buserelin, which is used to treat prostate or breast cancer. Buserelin in urine was determined by online combination of CZE-MS [57], off-line combination of preparative isotachophoresis (pITP) with MS [58], off-line pITP sample pretreatment before on-line CZE-MS [59] and pITP with off-line detection using surface-enhanced Raman spectroscopy (SERS) [60]. It was shown that pITP performed before analyses can significantly simplify complex matrix, and, due to its concentration power, pITP can consequently decrease the concentration LOD. Using pITP-SERS, the calculated LOD for buserelin was 62 pM (Table 2). As mentioned, buserelin is used to treat prostate or breast cancer. Pharmaceutical products for the treatment of these diseases are administered in the form of buserelin acetate. The high-precise determination of acetate, main constituent, and counterion in the pharmaceutical preparation buserelin acetate was carried out by ITP on the microchip with CD (Figure 6) [61]. The formulas of selected peptides discussed in Table 2 are shown in Figure 7.

### 3.3. Catecholamines

Catecholamines belong to biogenic amines formed by a catechol group and an ethylamine side chain, which can be further substituted with alkyl groups or a hydroxyl group [62]. Catecholamines are metabolites of the amino acid tyrosine. The rate-determining step of catecholamine biosynthesis is the transformation of tyrosine into dihydroxyphenyl alanine. This group of compounds plays an important role in metabolism and thermogenesis. Catecholamines stimulate not only oxygen synthesis, but also the exothermic consumption of glucose and fatty acids. Furthermore, catecholamines participate in the stimulation of glycogenesis and in the breakdown of triglycerides (lipolysis) [63]. Catecholamines are usually determined in body tissues and fluids, predominantly CSF, blood plasma, and urine. The analysis of such samples is of great importance for better understanding of sympathoadrenal function in laboratory animals and humans.

For the simultaneous determination of 12 biogenic amines, including the catecholamines dopamine (DA), epinephrine (EP) and norepinephrine (NE), a bioanalysis using CZE-MS^2^ was performed [64]. The lowest LOD among catecholamines found during the study was displayed for DA (0.0045 μg/mL or 0.029 μM), the highest LOD was demonstrated for EP (0.14 μg/mL, 0.76 μM). Linearity ranges of around two decadic orders were reported in the paper [64]. Simultaneous enantioseparation of catecholamines using a CZE-MS method was described by Sánchez-López et al. [65]. The achievement was made possible by using ESI-MS^2^ as a detector after a CZE separation: separate peaks were obtained for l- and d-NE, l- and d-EP in spiked plasma. The best enantioselectivity was obtained with addition of β-cyclodextrin derivatives to the BGE solution [65].

A very sensitive CZE-LIF method was developed for the quantitative analysis of neurotransmitter catecholamines and related amines in human urine and serum. The LOD value for DA was estimated at 30 pg/mL (0.2 nM). The separation was carried out with a sodium citrate buffer used as a BGE, in uncoated fused-silica capillaries, with LIF detection using a 473 nm laser diode as an excitation source [66].

LODs reported for other popular detection methods, such as AD or UV detection, are typically at least one decadic order higher. For example, Kolobova et al., using solid-phase extraction (SPE) as a pre-treatment method, dynamic coating, i.e., physical adsorption, of imidazolium liquids onto the inner surface of a fused-silica capillary, and a UV detector, reached a 1.3 ng/mL (7.6 nM) LOD for NE and a 0.3 ng/mL (2.0 nM) LOD for DA, 10 times higher than in the aforementioned study with LIF detection [67]. Another study describing similar conditions (SPE pre-treatment, sweeping of a capillary with sodium dodecyl sulfate, addition of ionic liquids to the BGE, UV detection) described LODs of 50 ng/mL for four catecholamine compounds [68].

SPE on micro- and nanoparticles is a promising relatively new method of sample pre-treatment in CE. It has been implemented for the determination of EP, NE, and other catecholamines in another study on enantioseparation by Wu et al. (magnetic particles coated with a copolymer of ethylenimine and 3,4-dihydroxyphenylalanine approximately 168  ±  4.3 nm in diameter) [69] and in a work by Polikarpova et al. (particles of a polystyrene-based cation exchanger 50 to 250 nm in diameter) [70]. Wu et al. claim that obtained LODs (400–600 pg/mL) are about ten times lower than LODs of chiral CE and report a linear range of more than one decadic order [69].

**Figure 8:**
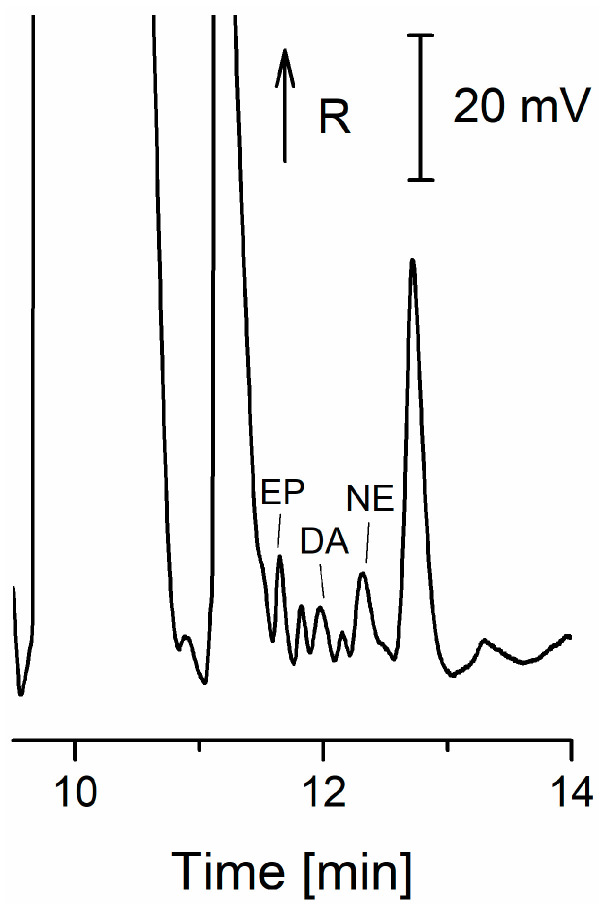
Electropherogram from microchip ITP-ZE separation of catecholamines in urine [72]. EP—epinephrine; DA—dopamine; NE—norepinephrine; R—resistance.

Roychoudhury et al. [71] reported MCE-AD separation and detection of DA, EP, and serotonin. Analytes were detected within 650 s without sample treatment, and the procedure was validated with spiked bovine serum samples. The combination of ITP-ZE on a microchip with CD was used for the determination of three catecholamines in urine (Figure 8). The calculated values for the LOD were in the range of 0.13–0.15 µM [72]. See Table 3 for the comparison of CE methods of catecholamine detection.

### 3.4. Drugs and Toxic Substances

Already a chapter in a book published more than 10 years ago [73] mentions electromigration techniques, especially CZE, as effective tools for the separation of charged compounds/drugs (especially acids and bases) due to the achievement of high resolution in a short time, which offers many advantages over other separation methods such as HPLC. Caslavska & Thormann highlight the advancements in CE-based chiral drug bioassays, focusing on their applications in pharmacokinetics, drug metabolism, and toxicological analysis, including key achievements and techniques for enantioselective determination of drugs and metabolites [74].

Selective serotonin reuptake inhibitors (SSRIs) are a prominent class of antidepressant drugs. It is recommended to monitor the concentrations of these medications in body fluids, usually urine, in order to ensure the effectiveness of treatment and prevent its negative side effects [75]. Murtada et al. used a composite of a styrene-vinylbenzene copolymer and multi-walled carbon nanotubes as a novel SPE sorbent for the determination of SSRIs in human urine. Linear ranges were approximately two decadic orders. The authors claim the method, among other advantages, allowed for recycling of the used nanotubes [76]. A different approach to the treatment of urine and plasma samples containing SSRIs as analytes was presented by Wang et al. They used dispersive LLE (acetone was chosen as a dispersive solvent and 1,1,2,2-tetrachloroethane as an extraction solvent) coupled with CZE-UV. The authors reported the method resulted in linear ranges exceeding two decadic orders for all analyzed SSRIs and LODs of 0.2 nM (0.06 ng/mL) for the two fluoxetine enantiomers in urine [77].

Similarly, the need to determine the level of antibiotics in human plasma [78] has also been discussed: for reasons of efficacy and prevention of the emergence of resistant strains and superbugs, for toxicological reasons, and also because detecting levels of antibiotics in blood paves the way to individualized treatment [79,80]. CZE-C^4^D and CZE with indirect UV detection were developed for determining fosfomycin in human plasma and micro dialysis samples, showing high sensitivity, reproducibility, and applicability in clinical studies, well-suited for clinical studies for the determination of the antibiotic in biological fluids without the need for extensive sample preparation. The LOD was between 0.6 and 2 μg/mL, depending on the matrix and the detection method. The yield determined with spiked samples was about 100%, the RSD% of independent determinations of Fosfomycin in triplicate after spiking Ringer’s solutions and plasma samples, respectively, was better than 8% [81]. Fu et al. [82] reported a simple and sensitive method for simultaneous electrogenerated-chemiluminescent (ECL) detection of quinolone residues in biological fluids, using CE, with successful application to pig urine after SPE. Enrofloxacin, levofloxacin, and ciprofloxacin can be assayed in the range of 3.0 × 10^−8^–5.0 × 10^−6^ g/mL within 10 min.

Addictology is a rapidly developing science. The need to identify addictive substances in the field leads to the application of ever-new methods [83]. Accurate determination of the concentration of addictive substances in body fluids after administration often becomes a life-saving measure [84,85,86]. A fast, precise (with the intra- and inter-day precision not exceeding 13%) and sensitive CZE method with multi-step sample pre-treatment involving SPE for forensic analysis of common narcotic drugs and toxic substances in human blood was proposed by Cui et al. [87]. The sensitivity of the implemented diode-array detector was higher than that of an ordinary UV detector. Linear ranges for the analytes exceeded two decadic orders [87].

Recent studies by Opekar & Tůma addressed the use of an electromembrane extraction (EME) probe online, coupled with CZE-C^4^D for the pre-treatment of biological samples [88]. EME was used to extract ketamine from untreated human urine. The LOD for ketamine was 1 µM [89]. A carbon nanotube-assisted electromembrane extraction (CNTs/EME) method coupled with CZE was developed for the pre-concentration and determination of cocaine in real samples, where CNTs embedded in the hollow fiber pores enhanced mass transfer and extraction efficiency at lower voltages compared to conventional EME. Under optimized conditions, the method achieved a low LOD (7.0 nM), high pre-concentration (178-fold), and good recovery (89%) [90]. The EME probe was also tested on methadone. Methadone was extracted into 3.0 M AcOH as acceptor. The enrichment factor was greater than 30, and the LOD was 0.7 μM [91]. In another study, β-cyclodextrin-modified gold nanoparticles (β-CD-AuNPs) were incorporated into a hollow fiber to enhance EME, which was coupled with CZE-UV for the determination of methadone in plasma. Compared with conventional EME, the β-CD-AuNPs/EME method achieved higher extraction efficiency, lower LODs (16 nM), improved recovery (68%), and a stronger pre-concentration factor (135) [92].

**Figure 9:**
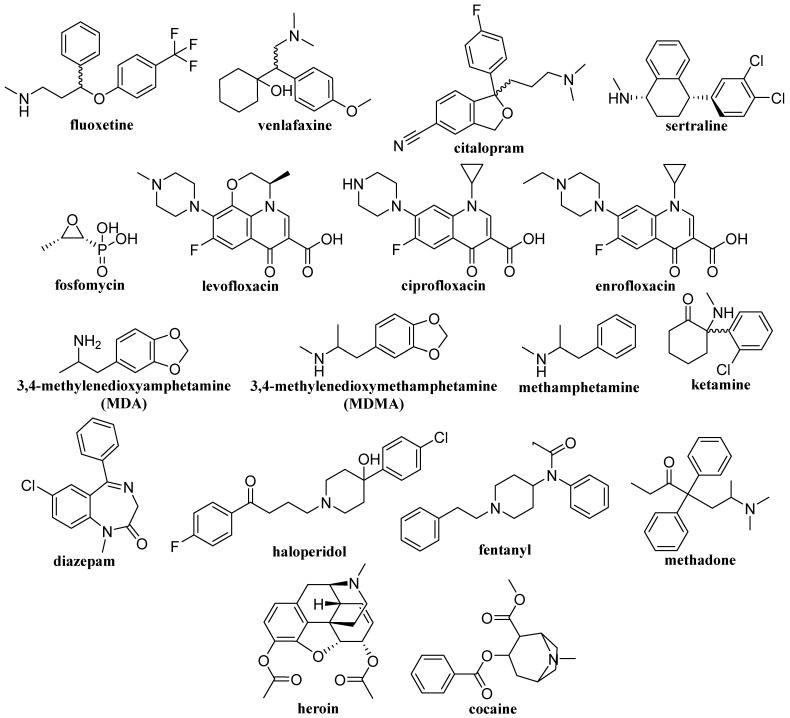
Structures of drugs and addictive substances.

The comparison of these methods’ analytical characteristics is presented in Table 4. The formulas of the drugs and addiction-relevant molecules discussed in this section are shown in Figure 9.

## 4. Critical View and Perspectives

Electromigration techniques, encompassing CZE and its variants such as ITP, MEKC, and MCE, have demonstrated substantial utility in the bioanalysis of body fluids, as reflected by their successful application in detecting amino acids, peptides, catecholamines, and drugs. These methods offer clear advantages over conventional chromatographic techniques, including high separation efficiency, short analysis times, minimal sample and reagent consumption, and cost-effectiveness. However, despite these strengths, several persistent challenges have hindered their broader adoption in clinical diagnostics and routine bioanalytical laboratories. They demonstrate notable resilience and practicality when compared with chromatographic and LC-MS approaches. Unlike chromatographic systems that depend on expensive columns, high solvent volumes, and frequent maintenance, CE operates with simple, reusable capillaries and consumes only microliters of electrolyte, greatly reducing cost and environmental impact. Its minimal sample and reagent requirements make it ideal for clinical laboratories handling limited or precious biological samples. Furthermore, the electrophoretic separation mechanism is highly reproducible, with capillary surface modifications and dynamic coatings enhancing robustness against matrix fouling. Although CE typically offers lower sensitivity than LC-MS, the integration of preconcentration steps (e.g., field-amplified stacking, on-line solid-phase or electromembrane extraction) and hyphenation with sensitive detectors such as LIF or MS has markedly closed this gap. Importantly, CE’s rapid analysis times, automation potential, and compatibility with microchip formats provide a practical, scalable alternative for high-throughput bioanalysis where chromatographic methods remain more labor-intensive and costlier. Consequently, CE-based platforms combine operational simplicity and sustainability with sufficient analytical performance, positioning them as viable complements—and in some contexts, replacements—for traditional LC-MS workflows in routine bioanalysis.

A critical assessment of current limitations and emerging trends provides insight into how these techniques may evolve toward greater reliability, automation, and sustainability [93,94,95].

### 4.1. Sensitivity and Matrix Complexity

One of the principal limitations of electromigration methods is their inherent sensitivity constraint when analyzing complex biological matrices such as urine, plasma, saliva, and CSF. The narrow capillary dimensions (25–100 µm i.d.) limit sample injection volumes to the nanoliter range, leading to relatively high LODs for low-abundance analytes that often exist at nanomolar or picomolar concentrations. Direct UV-visible detection, although simple and cost-efficient, typically requires chemical derivatization to enhance molar absorptivity, steps that can compromise sample integrity and extend analysis time. LIF substantially improves sensitivity (LOD < 5 nM for amino acids in urine) but depends on fluorogenic labeling, which may not be universal and can alter the analyte’s native properties. Conductivity-based methods such as C^4^D offer universality without derivatization but suffer from relatively higher LODs, restricting their use in trace-level biomarker studies [96,97,98].

Matrix effects further complicate CE separations. Biological fluids contain salts, proteins, and macromolecules that can adsorb onto the capillary wall, causing peak tailing, decreased resolution, and poor repeatability (often >5–10% RSD). For peptides and proteins, this adsorption problem is particularly acute, necessitating permanent (e.g., polyacrylamide, polyvinyl alcohol) or dynamic coatings, which increase cost and complexity but may not completely prevent fouling in viscous samples such as plasma [94,95]. In catecholamine and drug assays, variations in ionic strength or pH can disrupt EOF, leading to shifts in migration times and quantification errors. While extensive sample preparation (e.g., SPE, protein precipitation, or dialysis) can mitigate these effects, it also adds labor and introduces potential analyte loss. Hence, simplification and automation of sample treatment remain critical goals for clinical translation [93,97].

### 4.2. Hyphenation and Interface Limitations

The coupling of CE with MS represents one of the most significant advances in bioanalysis, providing superior selectivity and structural information for complex mixtures. Nevertheless, CE-MS interfaces are still technically demanding. Sheath-flow ESI designs dilute analyte zones, lowering sensitivity by up to threefold, whereas sheathless interfaces (despite offering 10–100 times higher sensitivity) often suffer from instability and compatibility issues with volatile background electrolytes. These limitations prevent their large-scale clinical applications, such as metabolomics or cancer biomarker profiling, where robustness and high throughput are essential [93,99].

### 4.3. Preconcentration, Miniaturization, and Greenness

Despite these limitations, electromigration techniques have a promising perspective for overcoming these problems and expanding into clinical diagnostics. Advanced online preconcentration techniques, including SPE coupled with CE-MS and field-amplified sample stacking, have achieved over 100-fold sensitivity enhancements (e.g., LOD 0.02 µg/mL for α-synuclein peptides in blood), allowing trace-level analysis in small sample volumes [100]. Aptamer-based affinity extraction and electroextraction further simplify sample handling and improve selectivity for peptide and catecholamine analysis in urine or plasma [101]. Parallel developments in MCE and 3D-printed devices are enabling rapid, portable point-of-care testing (such as the 3 min quantification of antibiotics in dried blood spots) while dramatically reducing reagent consumption and waste [95,102]. These trends align with the growing demand for miniaturized, cost-effective, and environmentally responsible analytical systems.

Electromigration techniques inherently support the principles of green analytical chemistry through minimal sample and solvent use. Current efforts toward eco-friendly operation include the development of water-based or biodegradable BGEs, recyclable polymer microchips, and energy-efficient high-voltage supplies [101], which can dramatically lower costs in clinical labs. Hyphenated systems like multisegment injection-CE-MS enable high-throughput screening, addressing throughput limitations. The convergence of CE with “green” microextraction approaches positions these methods as sustainable alternatives to traditional chromatographic systems, particularly for high-volume routine testing.

### 4.4. Automation, Artificial Intelligence, and Future Outlook

The future of electromigration bioanalysis lies in full automation, intelligent data processing, and compliance with regulatory standards. Integration of AI-driven algorithms for peak recognition, migration-time prediction, and quality-control validation will enhance reproducibility and throughput [103]. To achieve routine clinical implementation, standardized validation protocols and collaborative efforts among analytical chemists, clinicians, and instrument manufacturers are imperative.

Overcoming the intertwined challenges of sensitivity, matrix interference, and instrument robustness will transform electromigration techniques from specialized research tools into integral components of clinical and pharmaceutical laboratories. Continued synergy between CE-MS, microextraction, nanomaterials, and AI-assisted data processing promises real-time, multi-omics bioanalysis for personalized medicine. Expanding the analytical scope to less-explored biomolecules—such as lipids, carbohydrates, and exosomes—under native conditions will further enhance their biomedical relevance. Ultimately, through harmonized protocols, miniaturized platforms, and sustainable operation, electromigration techniques are poised to play a pivotal role in the next generation of clinical diagnostics and therapeutic monitoring.

## 5. Conclusions

Electromigration techniques (CE, ITP, MEKC, and MCE) are versatile and efficient tools for the bioanalysis of body fluids like urine, blood, saliva, and CSF. This work addresses their advantages in providing high-resolution separations of diverse analytes, ranging from amino acids and peptides as biomarkers for metabolic disorders to catecholamines for neurological assessments and drugs for therapeutic monitoring, while requiring minimal sample volumes and offering cost-effective alternatives to traditional chromatographic methods. Recent advancements, particularly in “hyphenated” systems like CE-MS and C^4^D, have enhanced sensitivity and specificity, enabling trace-level detection (e.g., LODs below 1 nM for amino acids) and facilitating applications in clinical diagnostics, forensic science, and pharmaceutical research. Despite ongoing challenges, such as matrix interferences in complex biological samples and the need for advanced sample preparation techniques like SPE or microextraction, these methods continue to develop. Innovations in capillary coatings, online preconcentration strategies (e.g., field-amplified sample stacking), and miniaturization have addressed limitations in reproducibility and throughput, paving the way for greener, more sustainable analyses with reduced solvent consumption. The integration of electromigration with emerging technologies, including microfluidic devices for point-of-care testing and AI-driven optimization for method validation, holds immense promise for overcoming current hurdles and expanding into multi-omics profiling.

Future efforts should focus on harmonized validation protocols, regulatory acceptance, and integration with advanced detection systems to fully establish electromigration techniques as sustainable and complementary platforms to conventional chromatographic bioanalysis. Innovations in capillary coatings, preconcentration, and miniaturization are expected to further enhance robustness and throughput, ensuring that CE-based methods continue to evolve toward broader clinical and pharmaceutical adoption.

## Figures and Tables

**Table 1:** Overview of CE methods for the determination of amino acids in body fluids: analytes, sample matrices, detection techniques, and LODs.

Analyte	Sample	Pre-Treatment	Method	LOD	Ref.
Gly, Ala, Ser, Val, Ile, Leu, Thr, Cys, Asp, Asn, Glu, Gln, Phe, Met, His, Arg, Tyr, Trp, Lys	Urine	Dilution in water	CZE-MS	1.62 μM Ala 0.51 μM Val 0.23 μM Ile 0.93 μM Leu 5.45 μM Gln 0.22 μM Arg	[27]
Lys, Gly, Ala, Cre, Val, Leu, Thr, Asn, Phe, Asp, Tyr	Capillary blood, blood plasma, tear fluid	Microdialysis	CZE-C^4^D	0.12 μM Lys 0.20 μM Gly, Val, Leu 0.19 μM Ala 0.17 μM Cre 0.24 μM Thr 0.26 μM Asn, Phe, Tyr 0.28 μM Asp	[28]
Leu, Pro, Phe, Tyr, Val, Thr, Met, Ser	Urine	Two-phase electroextraction	CZE-MS	5 nM Leu, Phe, Tyr, Thr 20 nM Pro, Val 100 nM Met, Ser	[31]
Ala, Val, Ile, Leu, Gln	Blood plasma	Mixing of unfrozen plasma with HCl-acidified ACN vortexing, centrifugation, supernatant collection	CZE-C^4^D	0.9 μM Ala, Gln 0.7 μM Val, Ile, Leu	[33]
Ala, Val, Ile, Leu, Gln	Blood plasma	Mixing with HCl-acidified ACN, vortexing	CZE-C^4^D	0.13 μM Ala, Val, Ile, Leu, 0.14 μM Gln	[34]
Val, Ile, Leu	Blood plasma	Dilution in ACN, filtration	CZE-C^4^D	0.4 μM Val, Ile, Leu	[35]
Ser, Asn, Thr, Gln, PEA, His, Gly, Glu, Ala, Asp, Tyr, Tau, Val, Met, Ile, Trp, Leu, Phe, Arg	Urine	Dilution with 20 mM NaOH and/or standard solutions	Flow-gate MEKC-LIF	<5.0 nM	[36]
Ala, *a*Ile Arg, Asn, Asp, *b*Ala, Car, Cit, Cys, GABA, Gln, Glu, Gly, 4Hyp, His, Ile, Leu, Lys, Met, Orn, Phe, Pro, Ser, Thr, Trp, Tyr, Val	Urine	Dilution	CZE-ESI-MS	0.63–29 μM	[37]
GABA, Gly, Glu	Microdialysates of periaqueductal gray matter	Large-volume sample stacking injection	CZE-C^4^D	9 nM GABA 10 nM Gly 15 nM Glu	[38]
Sar	Urine	Extraction and preconcentration with Hydro-MeC-MMIP	CZE-DAD	0.1 μM	[39]
Cys, Hcy	Blood plasma, urine	Derivatization and LLE	CZE-UV	0.2 μM Cys 0.5 μM Hcy	[40]
d-Trp, l-Trp	Urine	ITP treatment	ITP-CZE-UV	10 nM	[41]
DNP-Nleu, d-Trp, l-Trp	Urine	ITP treatment	ITP-CZE-UV	10 μM DNP-Nleu	[42]
LCAR, ALCAR	Breast milk	Precipitation, centrifugation, filtration	MCE(ZE-CD)	0.2 μM LCAR 0.6 μM ALCAR	[45]
3NT	Urine	Dilution	MCE(ZE-Vis)	4.2 nM	[46]
Hcy	Urine, saliva	SPME	MCE(ITP-ZE-CD)	1.4 μM	[47]

Sar—sarcosine; Hcy—homocysteine; PEA—phosphorylethanolamine; Car—carnosine; Cit—citrulline; Cre—creatine; GABA—γ-aminobutyric acid; 4Hyp—*trans*-4-hydroxyproline; Gln—glutamine; *a*Ile—alloisoleucine; *b*Ala—β-alanine; Orn—ornithine; 3NT—3-nitrotyrosine; LCAR—l-carnitine; ALCAR—acetyl-l-carnitine; DNP-Nleu—*N*-(2,4-dinitrophenyl)-l-norleucine.

**Table 2:** Summary of CE methods for peptides determination in body fluids: analytes, sample matrices, detection technique, and LODs.

Analyte	Sample	Pre-Treatment	Method	LOD	Ref.
Glutathione	Saliva	Hollow-fiber liquid-phase microextraction	CZE-AD	1.5 nM	[50]
Glutathione	Saliva	Dispersive micro-SPE	CZE-C^4^D	4.9 nM	[51]
Glutathione	Bovine blood	Masking of thiol group with iodoacetic acid, dilution, filtration	ITP-CD	10 µM	[56]
Dynorphin A endomorphin met-enkephalin	Blood plasma	SPE with nanoliter valve	CZE-MS	11 nM dynorphin A 8 nM endomorphin 2 nM met-enkephalin	[52]
Amyloid β peptide fragments	CSF	Dissolution in DMSO or 0.10–0.16% NH_4_OH; labeling with Fluoprobe 488 NHS (for ITP-LIF)	ITP-LIF, ITP-MS	0.03 nM Aβ 1–42 1 nM Aβ 1–40 0.05 nM Aβ 1–38	[54]
Pro-Hyp, Pro-Pro, Pro-Gly, Pro-Leu, Hyp, Pro	Urine	Derivatization with 4-fluoro-7-nitro-2,1,3-benzoxadiazole	CZE-LIF	19 nM Pro-Hyp 23 nM Pro-Pro, Pro-Gly 20 nM Pro-Leu 70 nM Hyp 55 nM Pro	[55]
Buserelin	Urine	Dilution	CZE-MS	0.4 µM	[57]
Buserelin	Urine	Dilution, pITP	pITP-MS	8 nM	[58]
Buserelin, anserine, carnosine	Urine	Dilution, pITP	pITP-CZE-MS	0.5 µM buserelin 0.7 µM anserine 0.8 µM carnosine	[59]
Buserelin	Urine	Dilution, pITP	pITP-SERS	62 pM	[60]

Aβ—Amyloid β.

**Table 3:** Overview of CE methods for the determination of catecholamines in body fluids: analytes, sample matrices, detection techniques, and LODs.

Analyte	Sample	Sample Pre-Treatment	Method	LOD	Ref.
DA, EP, NE	Urine	Centrifugation, filtration through 0.22 μm filters, and dilution by a 20% aqueous methanol solution	CZE-MS^2^	29 nM DA 760 nM EP 77 nM NE	[64]
DA, l-EP, l-NE	Rat blood plasma	ACN treatment, centrifugation, supernatant dilution with formic acid, sonication, filtration through 0.2 μm filters	CZE-ESI-MS^2^	77 nM DA 40 nM l-EP 150 nM l-NE	[65]
DA	Urine, blood serum	Centrifugation, methanol addition, repeated centrifugation, filtration through 0.45 μm filter (serum); centrifugation, filtration through 0.45 μm filter (urine)	CZE-LIF	0.2 nM	[66]
DA, EP, NE, NMN	Urine	SPE with an alumina sorbent, elution with 0.1 M acetic acid	MEKC-UV	2.0 nM DA 3.3 nM EP 7.6 nM NE 2.7 nM NMN	[67]
DA, EP, NE, NMN	Urine	EDTA addition, pH adjustment to 8.5, SPE on activated alumina sorbent, elution with acetic acid, drying, dissolution in acetic/formic buffer	MEKC-UV	300 nM	[68]
EP, NE, isoprenaline	Bovine blood, mouse blood	Magnetic SPE on microparticles coated with poly(3,4-dihydroxyphenylalanine)-polyethyleneimine copolymer	CZE-UV	2.0–3.0 nM	[69]
DA, EP, NE, NMN	Urine	SPE on nanoparticles of a polymeric cation exchanger	CZE-UV	20 nM DA 22 nM EP 24 nM NE 22 nM NMN	[70]
DA, EP	Spiked bovine serum	10-times dilution with 50 mM phosphate-buffer saline, pH 7.0, 0.9% NaCl	MCE(ZE-AD)	2.4 μM DA 3.6 μM EP	[71]
DA, EP, NE	Urine	Dilution, ITP treatment	MCE(ITP-ZE-CD)	130–150 nM	[72]

DA—dopamine; EP—epinephrine; NE—norepinephrine; NMN—normetanephrine; l-EP—l-epinephrine; l-NE—l-norepinephrine.

**Table 4:** Overview of CE methods for the determination of antidepressant and narcotic drugs/toxic agents in body fluids: analytes, sample matrices, detection techniques, and LODs.

Analyte	Sample	Pre-Treatment	Method	LOD	Ref.
SSRIs	Urine	Magnetic SPE	CZE-DAD	130 nM fluoxetine 58 nM venlafaxine 110 nM citalopram 46 nM sertraline	[76]
SSRIs	Urine, blood plasma	Ultrasound-assisted dispersive LLE	CZE-UV	0.2 nM (*R*)-fluoxetine, (*S*)-fluoxetine 0.3 nM (*R*)-norfluoxetine, (*S*)-norfluoxetine (in urine)	[77]
Fosfomycin	Plasma, microdialysates	Precipitation with methanol, centrifugation	CZE-indirect UV, CZE-C^4^D	4.5–14.5 µM	[81]
Enrofloxacin levofloxacin ciprofloxacin	Pig urine	SPE	CZE-ECL	28 nM enrofloxacin 28 nM levofloxacin 24 nM ciprofloxacin	[82]
Various narcotic drugs and toxic compounds	Whole human blood	Dissolution, SPE, evaporation, and redissolution of the eluate	CZE-DAD	110 nM 3,4-methylenedioxyamphetamine 103 nM 3,4-methylenedioxymethamphetamine 130 nM methamphetamine 84 nM ketamine 54 nM heroin 53 nM haloperidol 110 nM diazepam 89 nM fentanyl 99 nM cocaine	[87]
Ketamine	Urine	EME	CZE-C^4^D	1 µM	[89]
Cocaine	Urine	Carbon nanotubes assisted EME	CZE-UV	7.0 nM	[90]
Methadone	Urine, serum	EME	CZE-C^4^D	0.7 µM	[91]
Methadone	Plasma	β-cyclodextrin-modified Au nanoparticles/EME	CZE-UV	16 nM	[92]

## Data Availability

No new data were created or analyzed in this study. Data sharing is not applicable to this article.

## Abbreviations

The following abbreviations are used in this manuscript:

AD	amperometry detection
BGE	background electrolyte
CD	contact conductivity detection
CE	capillary electrophoresis
CSF	cerebrospinal fluid
CZE	capillary zone electrophoresis
C^4^D	capacitively coupled contactless conductivity detection
DAD	diode-array detection
EME	electromembrane extraction
ESI-MS^2^	a tandem mass spectrometer with electrospray ionization
ITP	isotachophoresis
LIF	laser-induced fluorescence detection
LLE	liquid–liquid extraction
LOD	limit of detection
MCE	microchip electrophoresis
MEKC	micellar electrokinetic chromatography
MS	mass spectrometry
UV	ultraviolet spectroscopy
SPE	solid-phase extraction
SPME	solid-phase microextraction
SSRIs	selective serotonin reuptake inhibitors
Vis	visible photometric detection
ZE	zone electrophoresis
